# A tribute to Eugene Braunwald (1929–2026)

**DOI:** 10.1172/JCI208900

**Published:** 2026-06-15

**Authors:** Douglas E. Vaughan

**Affiliations:** Northwestern University, Feinberg School of Medicine, Chicago, Illinois, USA.

With the passing of Eugene Braunwald on April 22, 2026, academic medicine has lost the most influential cardiologist of the modern era (Figure 1). Braunwald was born in Vienna, Austria, on August 15, 1929, into a life he would later describe as idyllic — excellent schools, piano lessons, a family at ease. On March 12, 1938, everything changed. The Anschluss brought the Nazi regime into Austria, and within days what had been a peaceful life became a fight for survival. The Braunwald family escaped at the end of July 1938, with close calls along the way, spending time in England before settling in New York.

He graduated first in his class from New York University School of Medicine in 1952, completed his residency at the Johns Hopkins Hospital, and in 1955 joined the National Heart Institute in Bethesda, Maryland, where he worked in the laboratory of Stanley Sarnoff studying the determinants of myocardial oxygen consumption. In 1961, he became Chief of the Cardiology Branch, and in 1966 Clinical Director of what is now the National Heart, Lung, and Blood Institute — one of the most consequential appointments in the history of American cardiology.

In 1958, a young man arrived at the National Heart Institute with a loud heart murmur, severe exertional symptoms, and a left ventricular outflow gradient of 74 mmHg. No one knew what to call what he had. When the cardiac surgeon Andrew Glenn Morrow opened the chest expecting a discrete fibrous obstruction, he found only hypertrophy. He performed a myectomy. The patient survived. Braunwald and Morrow kept investigating. Their 1959 paper in *Circulation* established that severe thickening of the heart’s walls was causing the obstruction (1). A landmark 1964 paper in the same journal defined hypertrophic cardiomyopathy as a unique clinical entity — now recognized as one of the most common genetic heart conditions in the world, affecting approximately 1 in 500 people, and a leading cause of sudden death in young athletes (2). The surgical technique they developed, septal myectomy, remains a cornerstone of treatment today.

His work at the NIH also produced one of the most consequential figures in the history of cardiology. In 1968, Braunwald and John Ross Jr. published in *Circulation* a landmark paper on the natural history of aortic stenosis that included a survival curve showing that patients remained stable during a prolonged latent period. Then, upon the onset of angina, syncope, or heart failure, the curve fell sharply: median survival of five years after angina, three years after syncope, and fewer than two years after heart failure (3). This figure established the symptom triad as the decisive trigger for intervention and governed the management of aortic stenosis for half a century. The corollary Braunwald articulated in a 1990 *JACC* editorial became one of the most quoted aphorisms in valvular heart disease — that the most common cause of sudden death in patients with aortic stenosis is premature surgery, a warning against operating on asymptomatic patients before the risk of waiting was clearly outweighed by the risk of the procedure (4). It was a judgment call encoded in a curve, and it shaped clinical practice worldwide.

A second transformative contribution challenged one of medicine’s most durable dogmas. For decades it was accepted that heart muscle destroyed by myocardial infarction died instantly, as if a light switched off. Working first in animal models and then in patients, his group demonstrated in a landmark 1971 paper that infarct size could be reduced by controlling the balance of oxygen supply and demand (5). This concept — that time costs muscle — is the intellectual foundation of every emergency cardiac protocol in use today: why catheterization laboratories are staffed around the clock, why door-to-balloon time is measured in every hospital, and why millions who survived heart attacks in the last half-century are alive to know it.

In 1984, Braunwald founded the Thrombolysis in Myocardial Infarction (TIMI) Study Group at Brigham and Women’s Hospital, which would go on to conduct more than 80 randomized trials enrolling over 450,000 patients. TIMI established tissue plasminogen activator as superior to older thrombolytics for treating acute MI and stroke, demonstrated that ACE inhibitors reduce postinfarction mortality by 19%, and built the evidence base for aggressive LDL reduction with statins. All three of these became worldwide standards of care.

As transformative as his science was, Braunwald’s influence as an institution builder may have been equally consequential. In 1968, he became founding Chairman of Medicine at the new University of California, San Diego. In 1972, he moved to Brigham and Women’s Hospital and Harvard Medical School, where he served as Chairman for 24 years — simultaneously at Beth Israel Hospital from 1980 to 1989 — overseeing a faculty that exceeded 1,000 physicians and scientists. He was a founding trustee and Chief Academic Officer of Partners HealthCare System, now Mass General Brigham.

Perhaps no measure of his legacy is more telling than the succession of the Hersey Professorship he held for more than two decades. His successor was Victor J. Dzau, who had trained under Braunwald as chief resident and junior faculty. Dzau’s foundational research on the renin-angiotensin system laid the scientific basis for ACE inhibitors — now among the most widely prescribed cardiovascular drugs in the world — and he went on to serve as President of the National Academy of Medicine. That Braunwald trained the man who succeeded him in his own chair and that this man led the highest body in American medicine, is not a coincidence. It is the compounding logic of great mentorship.

Dzau’s successor was Joseph Loscalzo, who has served as Chairman of Medicine and Physician-in-Chief at the Brigham since 2005. Internationally recognized for his work on the vascular biology of nitric oxide, redox biology, and network medicine, Loscalzo has published over 1,100 scientific articles, holds 32 patents, and has held continuous NIH funding for more than 35 years. The Hersey chair thus passed from Braunwald to one of his own trainees and then to one of the great cardiovascular physician-scientists of the next generation — each building on the standards and culture that Braunwald had established.

His personal research laboratory trained at least 130 fellows; the TIMI Study Group trained more than 50 research fellows now in 12 countries. Among those he mentored were Marc Pfeffer, Elliott Antman, Peter Libby, and Marc Sabatine, each of whom is a leader of consequence who has trained generations of their own. The lineage reads less like a list of colleagues than a genealogy of the field.

Braunwald served as President of the ASCI in 1974–1975. A conservative estimate places 25% to 40% of current ASCI members and a comparable share of the Association of American Physicians within his direct orbit of influence, a fraction that rises further when the trainees of his trainees are counted. *Science Watch* identified him as the most frequently cited author in all of cardiology, with an H-index of 228. His textbook, *Braunwald’s Heart Disease*, now in its 13th edition, has been the standard reference for every cardiologist trained in the last four decades (6). He coedited 12 editions of *Harrison’s Principles of Internal Medicine* and published more than 1,600 papers, the last in the final year of his life.

My own path to Dr. Braunwald began in west Texas. I trained in internal medicine under Donald Seldin at Parkland Hospital in Dallas — himself a titan of academic medicine and a man who knew better than most how to recognize and cultivate potential in others. It was Seldin who recommended me to Braunwald for cardiology training, and that recommendation opened a door that shaped everything that followed in my career. To have trained under both Seldin and Braunwald — contemporaries, intellectual giants, and men of extraordinary character — was a privilege I have never taken for granted.

What I found in Braunwald matched and exceeded every expectation. He was soft-spoken in manner but spellbinding at the podium, and finding a seat when he lectured at the great cardiovascular conventions of his era required arriving early and accepting that standing room might be all that remained. He was a master communicator, perhaps without equal in his ability to encapsulate a complex problem or a nascent concept with precision and clarity, reducing the tangled to the essential without losing any of the truth. He was driven and relentless in his pursuit of answers, yet patient in waiting for the science to provide them — unwilling to outrun the evidence, always pushing the frontier of cardiovascular medicine forward and always knowing where that frontier was. Those who worked alongside him were drawn forward by inspiration rather than compulsion. He commanded the highest standards and communicated them clearly enough that disappointing him was something people went to considerable lengths to avoid — not from fear (well maybe a little…), but from the particular weight of knowing that his expectations were grounded in a vision of what medicine could be at its best.

He was proud of his scientific accomplishments, but if you asked him what gave him the most joy, he spoke not of papers or trials but of the people whose lives and careers he had touched. When I left Boston to take a position at Vanderbilt, he came to my going-away party. Before the evening ended, he pulled me aside, looked me in the eye, and called me a “mensch.” I have received many honors in a long career. None has meant more.

He was the first adult cardiologist elected to the National Academy of Sciences. Harvard established the Eugene Braunwald Professorship in Medicine in 1996, and the American Heart Association created the Eugene Braunwald Academic Mentorship Award in 1999 — a fitting tribute for a man who considered mentorship the most rewarding work of his life. He received honorary doctorates from 24 universities on three continents, including from Oxford.

When the living Nobel Prize laureates in medicine were asked who had contributed the most to cardiology, every one of them named Eugene Braunwald. That unanimity — rare in any field, nearly inconceivable in science — was the verdict of the people best positioned to render it. He transformed heart disease from a near-certain death sentence into a condition that millions now live with and survive. He built departments, founded institutions, trained generations, and wrote the book from which those generations learned.

The boy who fled Vienna in 1938 with close calls and little else built something on these shores that will outlast any building bearing his name: a tradition of rigorous, humane, and consequential medicine, passed from mentor to trainee across seven decades, and now woven irrevocably into the fabric of American academic medicine.

A simple statement captures it best: cardiology before Braunwald and cardiology after Braunwald are not the same field.

## Figures and Tables

**Figure 1:**
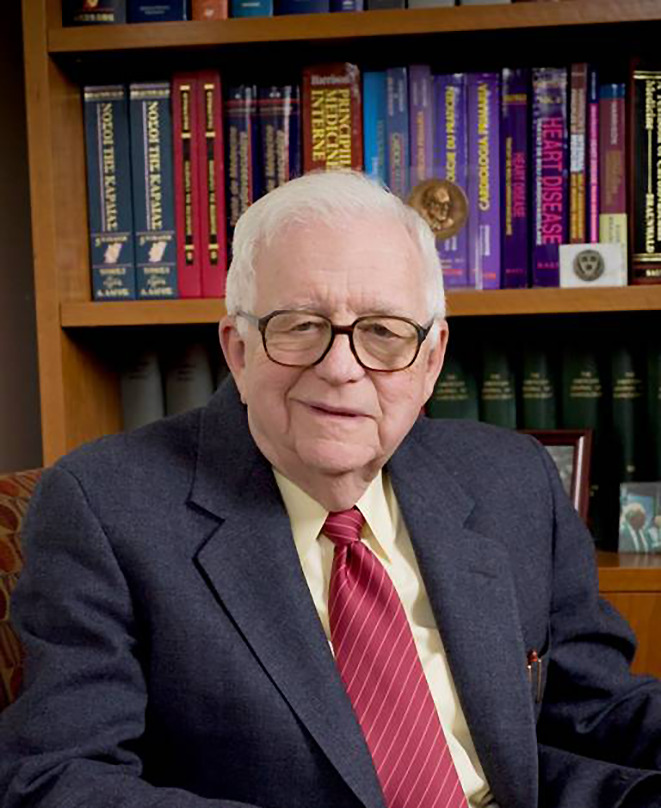
Eugene Braunwald. Image credit: Mass General Brigham.

## Version 1 06/15/2026

Electronic publication
